# Public acceptability of proposals to manage new takeaway food outlets near schools: cross-sectional analysis of the 2021 International Food Policy Study

**DOI:** 10.1080/23748834.2024.2336311

**Published:** 2024-04-19

**Authors:** Matthew Keeble, Jean Adams, Ben Amies-Cull, Michael Chang, Steven Cummins, Daniel Derbyshire, David Hammond, Suzan Hassan, Bochu Liu, Antonieta Medina-Lara, Oliver Mytton, John Rahilly, Nina Rogers, Bea Savory, Richard Smith, Claire Thompson, Christine M. White, Martin White, Thomas Burgoine

**Affiliations:** a MRC Epidemiology Unit, University of Cambridge School of Clinical Medicine, Cambridge, UK; b Nuffield Department of Primary Care Health Sciences, University of Oxford, Oxford, UK; c Department of Health and Social Care, Office for Health Improvement and Disparities, UK; d Population Health Innovation Lab, Department of Public Health, Environments & Society, Faculty of Public Health & Policy, London School of Tropical Hygiene and Medicine, London, UK; e Department of Public Health and Sport Sciences, Faculty of Health and Life Sciences, University of Exeter, Exeter, UK; f School of Public Sciences, Faculty of Health, University of Waterloo, Waterloo, Canada; g Department of Urban Planning, College of Architecture and Urban Planning, Tongji University, Shanghai, China; h Great Ormond Street Institute of Child Health, University College London, London, UK; i School of Health and Social Work, University of Hertfordshire, Hertfordshire, UK

**Keywords:** Exclusion zones, fast food, food environment, public acceptability, public support, takeaway food outlet, takeaway management zones around schools

## Abstract

Global trends indicate that takeaway food is commonly accessible in neighbourhood food environments. Local governments in England can use spatial planning to manage the opening of new takeaway outlets in ‘takeaway management zones around schools’ (known sometimes as ‘exclusion zones’). We analysed data from the 2021 International Food Policy Study to investigate public acceptability of takeaway management zones around schools. Among adults living in Great Britain (*n* = 3323), 50.8% supported, 8.9% opposed, and 37.3% were neutral about the adoption of these zones. Almost three-quarters (70.4%) believed that these zones would help young people to eat better. Among 16-17 year olds (*n* = 354), 33.3% agreed that young people would consume takeaway food less often if there were fewer takeaways near schools. Using adjusted logistic regression, we identified multiple correlates of public support for and perceived effectiveness of takeaway management zones. Odds of support were strongest among adults reporting that there were currently too many takeaways in their neighbourhood food environment (odds ratio: 2.32; 95% confidence intervals: 1.61, 3.35). High levels of support alongside limited opposition indicate that proposals for takeaway management zones around schools would not receive substantial public disapproval. Policy makers should not, therefore, use limited public support to rationalise policy inertia.

## Introduction

Evidence from multiple countries indicates that the number and density of hot food takeaway outlets (‘takeaways’) in neighbourhood food environments has increased since 2003 (Maguire *et al*. 2015, Taillie 2018, Needham *et al*. 2020, Pinho *et al*. 2020, Hobbs *et al*. 2021). This increase has coincided with greater normality and frequency of takeaway food consumption (Law *et al*. 2022). Takeaway food is often served in portions that exceed government guidelines for the consumption of energy, fat, salt and sugar (Jaworowska *et al*. 2014, Robinson *et al*. 2018, Huang *et al*. 2022). More frequent consumption of takeaway food has been associated with poorer diet quality (Barnes *et al*. 2016) and increased energy intake (Rosenheck 2008), both of which contribute to the prevalence of non-communicable diseases (Gesteiro *et al*. 2022). Previous reviews have reported positive associations between exposure to takeaways in the neighbourhood food environment and takeaway food consumption (Gesteiro *et al*. 2022, Wellard-Cole *et al*. 2022). Although the underlying evidence is equivocal, managing the number of takeaways in the neighbourhood food environment may prevent an increase in potential exposure, and therefore, benefit health at a population level (Sturm and Cohen 2009, Nykiforuk *et al*. 2018).

In England, opening a new takeaway requires approval through a process of applying for planning permission, with decisions made on applications in accordance with the national planning policy framework (Ministry of Housing Communities & Local Government 2018). As of 2018, around half of England’s 325 local authorities had adopted a place-based intervention through spatial planning that would enable them to manage the opening of new takeaways (Keeble *et al*. 2019a). Of these, 41 local authorities identified areas within specified distances from schools as ‘exclusion zones’. In part, this was a response to dietary patterns that include frequent takeaway food consumption and childhood obesity rates that have continued to rise in England since 2006 (Taher *et al*. 2018, NHS Digital 2021). Adoption and implementation of these zones does not mean that existing takeaways must close. Rather, local authorities seek to manage the opening of new takeaways in a designated area. Although ‘exclusion zone’ is a recognised term in England, we refer to these as ‘takeaway management zones around schools’ or ‘takeaway management zones’ to more accurately reflect the objective of this intervention.

Public acceptability of population health interventions contributes to their initial adoption and continued advocacy among local policymakers (Shill *et al*. 2012, Reynolds *et al*. 2020). Public acceptability can be inferred by assessing the extent to which proposals for interventions are supported (Diepeveen *et al*. 2013, Berinsky 2017). In 2018, 48.4% of adults in the United Kingdom (UK) supported takeaway management zone adoption when framed as a national-level intervention (Kwon *et al*. 2019). However, as it stands, adoption is locally determined, which is a distinction not previously considered. Public support for population health interventions, and the extent to which they are considered effective in achieving their stated aims (a further measure of public acceptability) can be influenced by how and why they are understood to operate (Pettigrew *et al*. 2023). Therefore, considering a single construct of public acceptability, as was previously the case, may have provided limited knowledge (Jepson *et al*. 2010, Eykelenboom *et al*. 2019). Evidence on how takeaway management zones around schools are perceived to operate in terms of their mechanism of change may help to address this previous limitation. Moreover, findings could inform public communications about the possible benefits of adopting takeaway management zones around schools. In turn, it might be possible to influence public acceptability, especially among less receptive population groups.

Evidence indicates that public acceptability of government-led population health interventions focused on obesity prevention varies by sociodemographic characteristics including age, sex and level of education, as well as existing food purchasing practices and household composition (Diepeveen *et al*. 2013, Quevedo *et al*. 2023). It may be that differences in levels of public acceptability reflect the extent to which individuals feel that the practices they want to engage in will be restricted should an intervention be adopted (Bos *et al*. 2013, Howse *et al*. 2022). For example, takeaway food consumption declines with age (Adams *et al*. 2015). Older individuals may, therefore, be more supportive of an intervention with a long-term aim of reducing takeaway food consumption because it would not affect them. Conversely, individuals who consume takeaway food more frequently may be less supportive (Hagmann *et al*. 2018). Understanding whether and how public acceptability of takeaway management zones around schools varies by individual-level sociodemographic characteristics may provide insights into the possibility that adoption will have differential and potentially unequal impacts across population groups. To our knowledge, this has not previously been investigated. Similarly, we are not aware of previous research investigating young peoples’ perspectives about takeaway management zones around schools, which is important given that improving the health of young people is often a core rationale for adoption of these zones.

In this study, we aimed to investigate the extent to which adults supported proposals for takeaway management zones around schools and viewed them as an effective way to help young people to eat better, and to identify the mechanisms through which they believed this could be achieved. Furthermore, we wanted to investigate whether and how public acceptability varied according to individual-level sociodemographic characteristics, measures of the neighbourhood food environment, and takeaway food purchasing practices and beliefs. Finally, we aimed to investigate what young people believed having fewer takeaways near schools could achieve.

## Methods

### Data

We analysed data from the International Food Policy Study (IFPS). This study consists of annual repeat cross-sectional online surveys conducted since 2017 in Australia, Canada, Mexico, the UK and the United States of America. Data collection methods have been described fully elsewhere (Hammond *et al*. 2022). Briefly, the IFPS comprises a youth survey (introduced in 2019; eligible respondents aged 10-17 years) and an adult survey (eligible respondents aged 18-100 years) where respondents answer questions about their sociodemographic characteristics, food related practices, and knowledge and beliefs about specific population health interventions. Respondents in the UK are recruited through the Nielsen Consumer Insights Global Panel and their partner panels. Email invitations with unique online survey access links are sent to a random sample of panellists after targeting for demographics. Adult respondents are asked to provide informed consent before survey completion. Parents or guardians of youth respondents are asked to provide consent for their child’s participation, and then answer questions about their household. Youth respondents are asked to provide informed assent before survey completion. The IFPS was reviewed by and received ethics clearance through a University of Waterloo Research Ethics Committee (ORE# 30829 and 41477).

### Sample

We analysed IFPS data from UK respondents, collected in November-December 2021 (Wave 5). There were 354 youth respondents aged 16-17 years and 4196 adult respondents (see supplementary material: Table S1 for a descriptive summary). We included all youth respondents (*n* = 354). We included all adult respondents with complete data for all measures of interest (*n* = 3323 (79.2%)).

### Outcome measures

Our outcome measures were levels of support for, and perceived effectiveness of, an intervention designed to stop new takeaways opening near schools, and levels of agreement that having fewer takeaways near schools could reduce how often young people consume takeaway food, make it easier to promote healthier food in schools, and to create healthier food environments near schools. Due to the sample size of youth respondents for inferential analysis (*n* = 354), our outcome measures apply to adult respondents only. The questions we included as outcome measures were not asked before 2021, so we were unable to include previous data from the IFPS. Table 1 presents the survey wording of our outcome measures and how we collapsed response options for statistical analysis.

**Table 1.** Survey questions and response options used as outcome measures to investigate public acceptability among adults towards takeaway management zones around schools^1^.

Concept	Question wording	Response options	Analytic categories ^2^
Adoption support ^3^	Some local councils are introducing regulations designed to stop new takeaways opening near schools. The aim is to help young people eat better If your local council was planning to introduce such regulations, would you support or oppose them?	Support	Support
		Neutral Oppose Don’t know Refuse to answer	Not support
Adoption effectiveness	*Preamble as above* How effective do you think these regulations will be?	Somewhat effective Mostly effective Very effective	Effective
		Not at all effective Don’t know Refuse to answer	Not effective
Ability for adoption to reduce takeaway food consumption	Please tell us whether you agree or disagree with the following: If there were fewer takeaways near schools: Young people would eat takeaway food less often.	Agree	Agree
		Neutral Disagree Don’t know Refuse to answer	Not agree
Ability for adoption to make it easier to promote healthier food in schools	*Preamble as above* Schools would find it easier to promote healthier eating.	Agree	Agree
		Neutral Disagree Don’t know Refuse to answer	Not agree
Ability for adoption to create healthier food environments near schools	*Preamble as above* Other types of healthier food outlets would be able to open.	Agree	Agree
		Neutral Disagree Don’t know Refuse to answer	Not agree

^1^Data were from Wave 5 of the International Food Policy Study, conducted November-December 2021. ^2^Analytic categories used in adjusted logistic regression, all response options reported as part of descriptive analysis.^3^ ‘local council’ = ‘local authority’.

### Covariates

During IFPS survey completion, adult and youth respondents answer conceptually similar questions, with variations in wording. Table 2 in the supplementary material lists the survey wording and all response options for the questions that we included as covariates. We provide an overview of these covariates, and how we collapsed response options for inferential analysis, in the sections that follow.

**Table 2.** Weighted summary statistics for adult respondents living in Great Britain in analytic sample (n = 3323). Data were from wave 5 of the International Food Policy Study, collected November-December 2021.

Measure	Category	*n* = 3323^1^
Age (years; mean (SD))	–	49.8 (17.0)
Age group (years)	18–29	556 (16.7%)
	30–44	802 (24.1%)
	45–59	851 (25.6%)
	60 or over	1114 (33.5%)
Sex	Male	1656 (49.8%)
	Female	1667 (50.2%)
Ethnic group identified with	Majority	2987 (89.9%)
	Minority	336 (10.1%)
Education level	Low	1630 (49.1%)
	Medium	719 (21.6%)
	High	973 (29.3%)
Ability to make ends meet	Not easy	1869 (56.2%)
	Easy	1454 (43.8%)
Child under 18 years in household	Yes	945 (28.4%)
Quartile (Q) of neighbourhood food outlet count	Q1 (0–9 outlets)	813 (24.5%)
	Q2 (10–25 outlets)	828 (24.9%)
	Q3 (26–55 outlets)	836 (25.1%)
	Q4 (56–581 outlets)	847 (25.5%)
Number of meals purchased from takeaways in the past week	0	1681 (50.6%)
	1	938 (28.2%)
	2 or more	704 (21.2%)
Perceived takeaway number appropriateness	Too many	1567 (47.2%)
	Too few	194 (5.8%)
	About the right number	1562 (47.0%)
Takeaways:		
Usually sell food that is affordable	Agree	1714 (51.6%)
	Disagree	375 (11.3%)
	Neutral	1209 (36.4%)
	Not stated	25 (0.8%)
Usually sell food that is poor quality	Agree	943 (28.4%)
	Disagree	782 (23.5%)
	Neutral	1569 (47.2%)
	Not stated	29 (0.9%)
Usually sell healthy food	Agree	453 (13.6%)
	Disagree	1862 (56.0%)
	Neutral	976 (29.4%)
	Not stated	33 (1.0%)
Cause litter, noise and smells	Agree	1942 (58.4%)
	Disagree	353 (10.6%)
	Neutral	998 (30.0%)
	Not stated	30 (0.9%)
Cause antisocial behaviour	Agree	907 (27.3%)
	Disagree	933 (28.1%)
	Neutral	1419 (42.7%)
	Not stated	65 (1.9%)
Contribute to the local economy	Agree	2033 (61.2%)
	Disagree	221 (6.6%)
	Neutral	1007 (30.3%)
	Not stated	62 (1.8%)
Support for management zone adoption^2^	Support	1687 (50.8%)
	Oppose	297 (8.9%)
	Neutral	1238 (37.3%)
	Not stated	101 (3.0%)
Perceived effectiveness of management zone adoption^2^	Somewhat effective	1646 (49.5%)
	Mostly effective	449 (13.5%)
	Very effective	246 (7.4%)
	Not effective	820 (24.7%)
	Not stated	161 (4.9%)
Following management zone adoption:^2^		
Takeaways would be replaced by healthier food outlets	Agree	1449 (43.6%)
	Disagree	499 (15.0%)
	Neutral	1268 (38.1%)
	Not stated	108 (3.3%)
It would be easier for schools to promote healthier food	Agree	1503 (45.2%)
	Disagree	585 (17.6%)
	Neutral	1148 (34.6%)
	Not stated	87 (2.6%)
Young people would eat takeaway food less often	Agree	1254 (37.7%)
	Disagree	807 (24.3%)
	Neutral	1153 (34.7%)
	Not stated	109 (3.3%)

^1^number (%) unless stated. May vary from total due to rounding after weighting. ‘Not stated’ = ‘Don’t know’ or ‘Refuse to answer’ responses. ^2^ ‘management zone’ = takeaway management zone around schools, sometimes referred to as ‘exclusion zones’ elsewhere.

#### Sociodemographic characteristics

We examined sociodemographic characteristics previously associated with takeaway food consumption, and differences in levels of acceptability toward the adoption of population health interventions (Janssen *et al*. 2018, Gesteiro *et al*. 2022, Barry *et al*. 2023, Toumpakari *et al*. 2023). Adult respondents reported their age in years (continuous), which we grouped (18-29 years, 30-44 years, 45-59 years, 60 years or over) based on evidence of a non-linear association between takeaway food consumption and age (Adams *et al*. 2015, Penney *et al*. 2017, 2018). Youth respondents reported whether they were 16 or 17 years of age, which we included as a binary measure. All respondents reported their ethnicity as the group that best described their racial or ethnic backgrounds. We categorised respondents as identifying with an ethnic ‘majority’ if they reported their ethnicity as White or an ethnic ‘minority’ for all other responses. Adult respondents reported their highest level of completed education. We classified them as having a: ‘low’ (high school completion or lower), ‘medium’ (some post-high school qualifications), or ‘high’ (university degree or higher) level of education. Adult respondents, and the parents or guardians of youth respondents (as previously described), reported their perceived income adequacy as their ability to make ends meet. We included this marker of socioeconomic position as a binary measure (‘not easy’, ‘easy’) (Chen *et al*. 2015, Gildner *et al*. 2016). Youth respondents also reported if their family had enough money to pay for the things they needed or not, which we included as a binary measure (‘not enough’, ‘enough’). Adult respondents reported the number of children aged under 18 years at home, which we included as a binary measure (‘no’, ‘yes’).

#### Food purchasing practices

Existing levels of takeaway food consumption may influence public acceptability of takeaway management zones around schools. Adult respondents reported the number of meals purchased in the week before survey completion from a ‘fast-food outlet, takeaway or café’. We categorised adult respondents as having purchased 0 meals, 1 meal or 2 or more meals, which reflected a right-skewed data distribution for this measure.

Youth respondents reported the number of days in the week before survey completion that they had purchased a meal from ‘restaurants, fast-food or takeaway outlets, food stands, or vending machines’. Response options ranged from 0 to 7 days. We included this as a binary measure (‘no’, ‘yes’). Youth respondents also reported if they had purchased ‘fast-food or takeaway food from a restaurant’ in the past 24-hours, which we included as a binary measure (‘no’, ‘yes’).

#### Neighbourhood food outlet access

Adult respondents reported their full residential postcode, which in England contain around 15 addresses on average meaning that they are relatively granular. We used Doogal (Doogal 2023) or GeoConvert (GeoConvert 2023), which are both geocoding platforms available online, to identify the coordinates of adult respondent postcodes. We mapped the postcode coordinates in a geographic information system (GIS); ArcGIS Pro version 10.7.1.

We used Ordnance Survey Points of Interest (OS POI) data collected between October and December 2021 (published in March 2022) to calculate neighbourhood food outlet access. This data is one of the most complete sources of food outlet location data available for Great Britain, and has been used for research purposes (Wilkins *et al*. 2017, 2019). Our use of OS POI data meant that we were unable to include adult respondents living in Northern Ireland (*n* = 70 (1.7% of 4196)). We refer to adults in our analytic sample as living in Great Britain to reflect that they lived across England, Scotland and Wales.

We extracted information from categories of OS POI data that include retailers from the out-of-home food sector: ‘Fast-food and takeaway outlets’ (food outlets serving food for consumption away from the premises), ‘Fast-food delivery services’ (food outlets serving food for delivery, not through online food delivery service platforms), ‘Fish and Chip shops’ (food outlets predominantly serving a specific type of cuisine for consumption away from the premises) and ‘Restaurants’ (food outlets serving food for consumption inside the premises that can also sell food to take away) (PointX 2006). We used coordinates supplied in OS POI data to map the location of food outlets in our GIS.

Finally, we counted the number of food outlets within a 1600 metre (approximately 1-mile) Euclidean (straight-line) radius of adult respondents’ postcodes to determine neighbourhood food outlet access. This distance is commonly used in neighbourhood food environment research, can reflect the spatial extent of food shopping practices, and be walked by an adult in 15-20 minutes (Wilkins *et al*. 2019). We categorised adult respondents into quartiles (Q) based on the number of food outlets within this radius, where those in Q1 had the lowest number. We used Q1 as the reference group throughout analyses.

#### Beliefs

Adult respondents reported how appropriate they believed that the number of takeaways they encountered on a daily basis was. Youth respondents answered the same question in the context of their school vicinity. We categorised all respondents as reporting that they encountered ‘too many’, ‘too few’, or ‘about the right number’.

In separate questions, adult respondents reported if they agreed or disagreed with, or were neutral about, statements that takeaways usually sell food that is affordable, healthy, and poor quality, and that takeaways cause litter, smells and noise, antisocial behaviour, and contribute to the economy. Youth respondents provided ‘true’ or ‘false’ responses to these questions. For all respondents, we included these questions as binary measures. For adults respondents, we combined disagree and neutral responses into a ‘not agree’ category.

Youth respondents were also asked what having fewer takeaways near school would mean (see supplementary material: Table S2 for statement wording). By checking a corresponding box for each statement, youth respondents indicated that they agreed. We included each statement as a dichotomous measure (‘checked’ or ‘not checked’).

### Analysis

Post-stratification sample weights constructed using a raking algorithm with population estimates derived from the census based on age group, sex, region, ethnicity, and education are available from the IFPS for adult and youth respondents, respectively (Hammond *et al*. 2022). We rescaled these sample weights to reflect the number of included respondents and applied them throughout analysis.

#### Statistical analysis

We used R (version 4.2.2, Vienna, Austria) to conduct descriptive and inferential analyses. Separately for adult and youth respondents, we quantified the measures of interest and report summary statistics for all included response options as part of our descriptive analysis. For adult respondents, we used separate logistic regression models to estimate associations between covariates and our outcome measures (see Table 1 for outcome measures and response option categorisation). In Model 1, we mutually adjusted for respondent sociodemographic characteristics and objective neighbourhood food outlet access. In Model 2, we additionally adjusted for beliefs about takeaways and takeaway food. We report adjusted odds ratios (OR) and 95% confidence intervals (CI) from Model 2 in the Results. We report the findings from Model 1 in the supplementary material (Table S3).

## Results

### International Food Policy Study respondent summary statistics

Table 2 summarises the characteristics of adult respondents in our analytical sample (n = 3323). Over half (50.8%) supported, a minority (8.9%) opposed, and more than one-third (37.3%) felt neutral about takeaway management zones around schools. Half (49.5%) reported that the zones would be somewhat effective at helping young people to eat better. Fewer reported that the zones would be mostly (13.5%) or very (7.4%) effective. A quarter (24.7%) reported that the zones would not be effective. Adult respondents typically agreed that having fewer takeaways near schools would allow healthier food outlets to open (43.6%) or make it easier for schools to promote healthier food (45.2%). Although 37.7% of adult respondents agreed that having fewer takeaways near schools would mean that young people would eat takeaway food less often, 24.3% disagreed with this statement.

Table 3 summarises youth respondent characteristics (n = 354). The majority of youth respondents (77.6%) disagreed with the statement that takeaways sell healthy food. Around half (49.6%) reported that takeaways provide young people with special offers, and are a place to hang out (52.8%). If there were fewer takeaways near schools, 49.4% of youth respondents reported that young people would eat more lunch from school and 58.7% reported that young people would not buy unhealthy food from other outlets. A third of youth respondents (33.3%) reported that young people would eat takeaway food less often, however, most did not report this (66.7%). Additionally, if there were fewer takeaways near schools, youth respondents typically reported that young people would not travel to takeaways further away from school (74.5%) nor have takeaway food delivered to school (87.8%).

**Table 3.** Weighted summary statistics for youth respondents living in the UK (n = 354). Data were from wave 5 of the International Food Policy Study, collected November-December 2021.

Measure	Category	*n*=354^1^
Age (years)	16	183 (51.7%)
	17	171 (48.3%)
Sex	Male	182 (51.4%)
	Female	172 (48.6%)
Ethnic group identified with	Majority	295 (83.3%)
	Minority	58 (16.5%)
	Not stated	1 (0.3%)
Parents ability to make ends meet^2^	Not easy	224 (63.2%)
	Easy	130 (36.8%)
Family income^3^	Not enough	92 (25.9%)
	Enough	262 (74.1%)
Purchase of food prepared out-of-home in the past week^4^	Yes	191 (53.9%)
	No	159 (44.9%)
	Not stated	4 (1.3%)
Consumption of takeaway food in past 24-hours	Yes	107 (30.2%)
	No	246 (69.4%)
	Not stated	1 (0.4%)
Perceived takeaway number appropriateness	Too many	56 (15.8%)
	Too few	66 (18.7%)
	About the right number	212 (59.8%)
	Don’t know	20 (5.6%)
Takeaways usually:		
Sell healthy food	True	69 (19.6%)
	False	275 (77.6%)
	Don’t know	10 (2.8%)
Have special offers for young people	True	175 (49.6%)
	False	145 (40.9%)
	Don’t know	34 (9.6%)
Usually sell food that is low-cost	True	207 (58.5%)
	False	128 (36.0%)
	Don’t know	19 (5.4%)
Are an important place for young people to hang out	True	187 (52.8%)
	False	132 (37.3%)
	Don’t know	35 (9.9%)
If there were fewer takeaways near schools, young people would:		
Be more likely to eat lunch in the school canteen	Checked	175 (49.4%)
	Not checked	179 (50.6%)
Buy unhealthy food from other types of food outlets	Checked	146 (41.3%)
	Not checked	208 (58.7%)
Eat takeaway food less often	Checked	118 (33.3%)
	Not checked	236 (66.7%)
Travel to takeaways further away from school	Checked	90 (25.5%)
	Not checked	264 (74.5%)
Have takeaway food delivered to school	Checked	43 (12.2%)
	Not checked	311 (87.8%)
Unclear what the effect of adoption would be^6^	Checked	33 (9.3%)
	Not checked	321 (90.7%)

^1^number (%) unless stated. May vary from total due to rounding after weighting. ^2^Youth respondent’s parent/guardian perceived ability to make ends meet. ^3^Family income answered by youth respondent. ^4^‘out-of-home’ = from ‘restaurants, fast food or take-away places, food stands, or vending machines’. ^5^‘Checked’ = agree, ‘Not checked’ = disagree. ^6^‘Don’t know’ or ‘Refuse to answer’ responses, only available when no other responses checked.

### Covariates associated with measures of public acceptability

Table 4 presents the findings from our logistic regression analyses for adult respondents after mutual adjustment for the listed sociodemographic characteristics, measures of the neighbourhood food environment, and takeaway food purchasing practices and beliefs.

**Table 4.** Adjusted Odds Ratios (OR) and 95% confidence intervals (CI) of sociodemographic characteristics, measures of the neighbourhood food environment, and takeaway food purchasing practices and beliefs associated with public acceptability of takeaway management zones among adults in Great Britain in 2021 (n = 3323)^1^. Data were from wave 5 of the International Food Policy Study, collected November-December 2021, analysed using logistic regression.

	Support for adoption		Perceived effectiveness		Healthier outlets able to open		Healthier school food promotion		Less takeaway food consumption	
Measure^2^	OR	95% CI	OR	95% CI	OR	95% CI	OR	95% CI	OR	95% CI
Age group (years); 18-29 = ref										
30-44	1.08	0.80, 1.45	**0.51**	**0.37, 0.71**	1.13	0.85, 1.49	0.80	0.60, 1.07	1.13	0.85, 1.50
45-59	**1.51**	**1.11, 2.07**	**0.42**	**0.30, 0.59**	1.00	0.74, 1.34	0.93	0.69, 1.26	1.16	0.85, 1.58
60 or over	**2.07**	**1.51, 2.83**	**0.46**	**0.32, 0.64**	1.02	0.75, 1.38	1.19	0.88, 1.62	1.29	0.95, 1.77
Sex; Female (vs Male)	1.17	0.98, 1.39	1.10	0.92, 1.32	**1.29**	**1.09, 1.52**	**1.29**	**1.08, 1.52**	0.99	0.83, 1.18
Ethnic group identified with; Minority (vs Majority)	1.31	0.97, 1.77	**1.57**	**1.10, 2.24**	1.28	0.97, 1.70	**1.46**	**1.10, 1.94**	**1.43**	**1.07, 1.92**
Education level; Low = ref										
Medium	1.25	1.00, 1.55	1.03	0.83, 1.28	0.92	0.75, 1.13	0.97	0.79, 1.19	1.04	0.84, 1.29
High	**1.57**	**1.29, 1.92**	1.17	0.96, 1.42	0.88	0.73, 1.06	1.09	0.90, 1.32	1.08	0.90, 1.31
Ability to make ends meet; Easy (vs Not easy)	**1.36**	**1.13, 1.63**	1.16	0.97, 1.40	1.01	0.84, 1.20	1.03	0.86, 1.22	1.08	0.91, 1.29
Child under 18 years in household; Yes (vs No)	0.97	0.77, 1.24	**1.47**	**1.14, 1.89**	0.95	0.76, 1.19	1.22	0.98, 1.53	1.05	0.83, 1.32
Quartile (Q) of neighbourhood food outlet access (count); Q1 (0-9 outlets) = ref										
Q2 (10-25 outlets)	0.80	0.63, 1.02	1.22	0.96, 1.55	1.00	0.79, 1.26	0.95	0.76, 1.20	0.92	0.73, 1.17
Q3 (26-55 outlets)	0.89	0.69, 1.14	1.05	0.83, 1.35	1.01	0.80, 1.28	1.00	0.79, 1.27	1.10	0.87, 1.40
Q4 (56-581 outlets)	0.78	0.61, 1.01	1.09	0.85, 1.41	0.98	0.77, 1.24	1.07	0.84, 1.36	1.10	0.86, 1.40
Meals purchased from takeaways in the past week (count); 0 = ref										
1	0.90	0.72, 1.13	1.04	0.83, 1.31	1.14	0.93, 1.41	1.18	0.95, 1.46	1.24	1.00, 1.53
2 or more	0.80	0.62, 1.04	1.14	0.87, 1.49	1.23	0.96, 1.57	1.05	0.82, 1.35	1.16	0.90, 1.48
Perceived takeaway number appropriateness; Too few = ref										
Too many	**2.32**	**1.61, 3.35**	1.29	0.87, 1.92	1.01	0.70, 1.46	0.94	0.65, 1.35	1.05	0.73, 1.50
About the right number	0.90	0.63, 1.29	1.40	0.95, 2.08	0.78	0.54, 1.11	**0.63**	**0.44, 0.89**	0.62	0.44, 0.88
Takeaways:										
Usually sell affordable food; Agree (vs Not agree)	**1.26**	**1.05, 1.52**	1.03	0.86, 1.24	**1.65**	**1.38, 1.96**	**1.69**	**1.42, 2.01**	**1.29**	**1.08, 1.54**
Usually sell poor quality food; Agree (vs Not agree)	**1.39**	**1.12, 1.73**	1.18	0.95, 1.46	**1.41**	**1.15, 1.73**	1.23	1.00, 1.50	**1.37**	**1.13, 1.67**
Usually sell healthy food; Agree (vs Not agree)	**1.37**	**1.05, 1.79**	1.15	0.86, 1.54	**1.42**	**1.10, 1.83**	**1.37**	**1.06, 1.76**	**1.62**	**1.27, 2.07**
Cause litter, noise and smells; Agree (vs Not agree)	**1.91**	**1.56, 2.33**	1.19	0.97, 1.47	**1.76**	**1.44, 2.15**	**2.02**	**1.66, 2.47**	**1.72**	**1.40, 2.12**
Cause antisocial behaviour; Agree (vs Not agree)	**1.33**	**1.07, 1.66**	1.14	0.92, 1.42	**1.51**	**1.23, 1.85**	**1.70**	**1.38, 2.10**	**1.36**	**1.11, 1.67**
Contribute to the economy; Agree (vs Not agree)	**1.37**	**1.13, 1.67**	**0.69**	**0.57, 0.84**	**1.21**	**1.01, 1.46**	1.12	0.93, 1.35	1.19	0.98, 1.43

^1^All results are adjusted for all other measures listed. Bold = significant at the *p* < 0.05 level. ^2^‘ref’ = reference category.

Compared with adult respondents aged 18-29 years, those aged 45-59 years (OR: 1.51; 95% CI: 1.11, 2.07), or 60 years or over (OR: 2.07; 95% CI: 1.51, 2.83) had greater odds of supporting takeaway management zone adoption. Those with a high rather than low level of education (OR: 1.57; 95% CI: 1.29, 1.92), those who found it easy to make ends meet (OR: 1.36; 95% CI: 1.13, 1.63), and those who reported that they encountered too many takeaways on a daily basis, rather than too few, also had greater odds of support (OR: 2.32; 95% CI: 1.61, 3.35). Beliefs about takeaways and takeaway food were often associated with greater odds of support. This association was strongest among adult respondents who agreed that takeaways cause litter, noise and smells (OR: 1.91; 95% CI: 1.56, 2.33).

Adult respondents who identified with an ethnic minority (OR: 1.57; 95% CI: 1.10, 2.24), and those who lived with a child under 18 years of age (OR: 1.47; 95% CI: 1.14, 1.89) had greater odds of reporting that takeaway management zones would be effective in helping young people to eat better. Age was inversely associated with perceived effectiveness, and those who agreed that takeaways contribute to the economy had lower odds of perceived effectiveness (OR: 0.69; 95% CI: 0.57, 0.84).

Adult respondents who were female had greater odds of agreeing that if there were fewer takeaways near schools then healthier food outlets could open (OR: 1.29; 95% CI: 1.09, 1.52). Beliefs about takeaways and takeaway food were each separately associated with greater odds of agreeing that healthier food outlets could open.

Adult respondents who were female (OR: 1.29; 95% CI: 1.08, 1.52), and those who identified with an ethnic minority (OR: 1.46; 95% CI: 1.10, 1.94) had greater odds of reporting that if there were fewer takeaways near schools then schools would find it easier to promote healthier eating. We also observed this association for several of the beliefs about takeaways and takeaway food. The strongest association was among adult respondents who agreed that takeaways cause litter, noise and smells (OR: 2.02; 95% CI: 1.66, 2.47). Those who believed that they encountered an appropriate number of takeaways on a daily basis, rather than too few, had lower odds of reporting that if there were fewer takeaways near schools then it would be easier for schools to promote healthier eating (OR: 0.63; 95% CI: 0.44, 0.89).

Adult respondents who identified with an ethnic minority had greater odds of reporting that if there were fewer takeaways near schools then young people would eat takeaway food less often (OR: 1.43; 95% CI: 1.07, 1.92). Adult respondents who agreed that takeaways cause litter, noise and smells (OR: 1.72; 95% CI: 1.40, 2.12), sell healthy food (OR: 1.62; 95% CI: 1.27, 2.07) sell poor quality food (OR: 1.37; 95% CI: 1.13, 1.67), cause antisocial behaviour (OR: 1.36; 95% CI: 1.11, 1.67) or sell affordable food (OR: 1.29, 95% CI: 1.08, 1.54) also had greater odds.

## Discussion

### Summary of findings

We analysed cross-sectional data collected in 2021 from adults living in Great Britain and young people aged 16-17 years living in the UK to investigate public acceptability of takeaway management zones around schools. Half of the adults in our sample supported the adoption of these zones. A minority opposed adoption and over one-third were neutral about this. Almost three quarters of the adults in our sample agreed that takeaway management zones around schools would be either somewhat, mostly, or very effective at helping young people to eat better, while a quarter stated that they would not be effective.

We identified that several sociodemographic characteristics, measures of the neighbourhood food environment, and takeaway food purchasing practices and beliefs were associated with measures of public acceptability of takeaway management zones around schools. The strength of association varied. Reporting that there were too many takeaways in the neighbourhood food environment was associated with support for takeaway management zones around schools. Living with a child aged under 18 years and identifying with an ethnic minority were each associated with greater perceived effectiveness of takeaway management zones around schools to help young people to eat better. Being older was associated with lower levels of perceived effectiveness, as was a belief that takeaways contribute to the economy. Being female was associated with agreeing that having fewer takeaways near schools would allow other types of healthier food outlets to open, and that it would make it easier for schools to promote healthier food. Beliefs about takeaways and takeaway food were often associated with support for takeaway management zones around schools, perceived effectiveness at helping young people to eat better, and agreeing that takeaways near schools would allow other types of healthier food outlets to open, allow schools to promote healthier food more easily, and mean that young people would eat takeaway food less often. The strength of association was typically strongest for less favourable beliefs, such as that takeaways cause antisocial behaviour and litter, noise, and smells.

Among the 16 and 17 year olds in our sample, around half reported that takeaways have special offers aimed at young people and are an important place for socialising. Over three-quarters reported that takeaways sell food that is unhealthy, and over half reported that the food is low-cost. If there were fewer takeaways near schools, around half of our sample reported that young people would eat more food in school and one-in-three reported that young people would eat takeaway food less often. Additionally, most reported that young people would not buy unhealthy food from other types of food outlet, travel to takeaways further away from school, or have food delivered to school.

### Strengths and limitations

In the IFPS, respondents are not recruited using probability-based sampling. As a result, our sample was not necessarily nationally representative. However, we used sampling weights, which can help to address sampling bias. As we analysed cross-sectional data, we cannot draw conclusions on the direction of causation between putative exposure and outcome measures. Furthermore, there are differences in planning systems and planning laws across England, Scotland and Wales, and also internationally (Chang *et al*. 2022). As a result, our findings are most relevant to England and may not be fully generalizable to all contexts.

During the IFPS survey, respondents were asked about hypothetical takeaway management zones around schools. This means that our findings do not necessarily reflect confirmed proposals. We also do not know whether respondents lived in an area with an existing takeaway management zone, and if so, their knowledge of and exposure to this. Additionally, we cannot rule out that respondents may have interpreted the zones as a way to close existing takeaways, as an outright ban on all new takeaways or as a way to prevent new chain fast-food restaurants from opening. Each of which would not be the case. However, we co-produced survey questions with members of the public, which might have helped to minimise possible misinterpretation.

In analyses, we included data for a range of sociodemographic characteristics, and takeaway food purchasing practices and beliefs based on previous evidence related to takeaway food consumption and public acceptability of population health intervention adoption (Janssen *et al*. 2018, Gesteiro *et al*. 2022, Barry *et al*. 2023, Toumpakari *et al*. 2023). Doing so allowed us to investigate multiple plausible associations. We also included objective and subjective neighbourhood food environment measures. With regard to the latter, our conclusions are based on IFPS respondent perspectives rather than researcher-defined neighbourhood food environments that do not necessarily always reflect food purchasing practices (Lytle 2009, Wilkins *et al*. 2019, Christensen *et al*. 2021). Nevertheless, we did not investigate latent factors such as relationships with takeaway owners, which may influence opinions about takeaways and in turn, targeted interventions.

### Contribution to knowledge

Half of the adults in our sample supported proposals for takeaway management zones around schools. A previous analysis of IFPS data that were collected in 2018 estimated that 48.4% of adults in the UK supported adoption when framed as a national government-led intervention (rather than local government-led as in 2021) (Kwon *et al*. 2019). Together, this evidence suggests that proposals to manage the number of takeaways opening near schools would be well supported by adults in the UK. This conclusion is further supported by our finding that one-in-three adults were neutral about proposals, and only a minority explicitly opposed them. To our knowledge, we are the first to investigate support for locally proposed takeaway management zones around schools. Although levels of support are already high, it is possible that, over time, they will further increase (Diepeveen *et al*. 2013). Therefore, our findings can be seen as a baseline to assess any future changes against.

The majority of adults in our sample reported that takeaway management zones around schools would be at least somewhat effective in helping young people to eat better. From a population health perspective, it seems encouraging that members of the public viewed this structural intervention as effective because it suggests an awareness about the possibility that the food environment could influence takeaway food consumption. Elsewhere, adults held a similar perspective about how the food environment could influence food purchasing practices (Grunseit *et al*. 2019, Neve and Isaacs 2021). Nevertheless, a quarter of the adults in our sample did not believe that takeaway management zones would be effective. This belief might reflect that takeaways are one component of neighbourhood food environments and that energy-dense and nutrient-poor food can be purchased from non-takeaway food retailers, including supermarkets (Wellard-Cole *et al*. 2022, Rose *et al*. 2022), and through digital purchasing formats including online food delivery service platforms (Keeble *et al*. 2021, 2022).

Adults in our sample often agreed that if there were fewer takeaways near schools then it would allow other types of healthier food outlets to open, allow schools to promote healthier food more easily, and mean that young people would eat takeaway food less often. However, these beliefs might not necessarily be directly attributable to takeaway management zones around schools. Whilst schools could feel empowered and incentivised to promote healthier food (Pillay *et al*. 2022), this is likely to be an indirect outcome of adoption, since these zones are not necessarily intended to make it easier for schools to promote healthier food. It might be that the intent and scope of takeaway management zones around schools needs to be refined and clarified. Given the influence of intervention framing on levels of public acceptability (McIntyre 2020, Koon and Marten 2023), clarification from local authorities in their public communications would go some way to addressing any potential misunderstanding.

Adults in our sample who reported that there were too many existing takeaways in the neighbourhood food environment, had greater odds of support for takeaway management zones around schools. As discussed, one possible explanation for this finding is a belief that the food environment can influence takeaway food purchasing and consumption. However, we did not identify an association between support and objective measurement of the number of takeaways around the home address. We have previously identified an association between the number of takeaways in a local authority boundary and takeaway management zone adoption (Keeble *et al*. 2019b). Therefore, despite our finding, the existing number of takeaways in a local authority continues to have relevance for policy adoption.

Older adults, those who had completed a university degree, and those with a greater ability to make ends meet were more supportive of takeaway management zones around schools. An individual’s personal context and the extent to which they believe they would be impacted by the intervention may have influenced observed associations. Our finding in relation to age speaks to previous evidence of decreased takeaway food consumption (Janssen *et al*. 2018, Mills *et al*. 2018), and a reduced influence of takeaway exposure on food purchasing practices (van Erpecum *et al*. 2023), among older adults. Higher levels of education are often correlated with income, and in turn, can influence perceived income adequacy (Galobardes *et al*. 2006a, 2006b, 2007, Gildner *et al*. 2016). It is feasible that those who find it easier to make ends meet have the flexibility to choose between purchasing takeaway food and eating out-of-home but inside of restaurants. In turn, stopping new takeaways from opening might be less likely to influence choices among certain population groups, leading to the greater levels of support that we observed.

Less favourable beliefs about takeaways and the food they sell were consistently associated with the outcome measures we investigated. Support for adoption was particularly evident among individuals who believed that takeaways cause litter, noise and smells. Takeaway management zones can be used to decide if, where, and when new takeaways would be allowed to open. As such, the adoption of these zones also provide the opportunity to direct resources to control issues such as litter, noise and smells. Our findings speak to the broader societal benefits that may come about as a result of adopting takeaway management zones around schools. These benefits are not necessarily about the diet and health of young people. In 2018, we demonstrated that less favourable elements of takeaways are considered when determining the appropriateness of takeaway planning applications (Keeble *et al*. 2019a). It might be that policy advocates can use these broader societal benefits as a leverage point since they may be less subject to opposition from critics compared with diet and health based rationales (Nixon *et al*. 2015). Therefore, rather than focusing only on takeaway food consumption, other considerations like antisocial behaviour and litter, noise, and smells can and should be emphasised as part of a broader approach to population health improvement (Cohen 2021, Felmingham *et al*. 2023).

Living with a child under 18 years of age was not associated with support for takeaway management zones around schools. It was, however, associated with the belief that they would be effective in helping young people to eat better. In a sample of mothers in Australia, there was support for a similar intervention, with variation according to levels of concern for their own child’s susceptibility to being overweight or obese but not the susceptibility of other children (Esdaile *et al*. 2021). Given that around 70% of the adults in our sample did not live with a child under 18 years of age, it is possible that concerns about takeaway consumption among young people were not a priority for our respondents, which in turn influenced the extent to which they supported takeaway management zones.

Most young people in our sample reported that takeaways offer a place to hang out and that they sell low-cost, unhealthy food. Our findings are aligned with the views of other young people living in the UK (Macdiarmid *et al*. 2015, Wills *et al*. 2016, Caraher *et al*. 2016, Burningham and Venn 2021, Shaw *et al*. 2023). That is, the ‘takeaway experience’ can outweigh an awareness that the foods sold are often classified as less-healthy than others (Jackson and Viehoff 2016), and that purchasing healthier takeaway food items when available does not conform to social expectations (Wills *et al*. 2016). Importantly, young people did not typically believe that having fewer takeaways near schools would lead to less takeaway food consumption. This finding emphasises that existing takeaways would remain accessible despite adoption or implementation of takeaway management zones around schools, and underlines a need to consider this intervention as one part of interconnected and long-term efforts to prevent diet-related ill-health among future generations. Nevertheless, from a population health perspective, it is encouraging that having fewer takeaways near schools (as a long term outcome of takeaway management zone adoption) would not necessarily prompt young people to travel to takeaways further away from school, which is a plausible way that this intervention could be undermined (Keeble *et al*. 2021).

### Policy and practice implications

Our findings add to a body of evidence regarding takeaways and the neighbourhood food environment that has accumulated over time. We identified high levels of support for, and low levels of explicit opposition to, proposals for takeaway management zones around schools. We also identified that these zones were perceived to be effective at helping young people to eat better. As this intervention is already acceptable to the public, establishing support at a later point is not necessary. Our findings therefore provide reassurance to policy makers that proposals to adopt takeaway management zones around schools would not necessarily lead to public resistance (John *et al*. 2023).

### Unanswered questions and future research

The health of young people is one rationale for takeaway management zones around schools. Further qualitative research is needed to investigate the views of young people regarding these zones in terms of their scope and possible strengths and limitations. The findings from this research might contribute to future intervention development, monitoring, and evaluation, and be especially important in the context of increased access to and availability of takeaway food through alternative purchasing formats including online food delivery service platforms (Keeble *et al*. 2023, Hoenink *et al*. 2023, Kalbus *et al*. 2023). The findings would also be complemented by a better understanding of why certain groups of adults appear to support proposals yet not believe they would be effective at helping young people to eat better.

Local authorities in England have already adopted takeaway management zones around schools. The extent to which public acceptability influenced their adoption is unclear. A better understanding of this would inform the possible need for a concerted effort to further strengthen the levels of public acceptability that we have identified. This understanding could help to influence local policy making processes and shift possible political perspectives that this intervention is useful but not a priority.

## Conclusions

In 2021, a sample of 3323 adults living in Great Britain responded to the International Food Policy Study. Of these respondents, 50.8% (*n* = 1687) explicitly supported the adoption of takeaway management zones around schools (referred to elsewhere as ‘exclusion zones’). Furthermore, more than one in three (*n* = 1238; 37.3%) reported that they did not have a strong opinion, whilst a minority (*n* = 297; 8.9%) explicitly reported that they were against the adoption of these zones. Adult respondents in our sample also typically agreed that these zones would be effective at helping young people to eat better and that if there were fewer takeaways near schools, healthier food outlets could open, it would be easier for schools to promote healthier food, and young people would eat takeaway food less often. A range of sociodemographic characteristics, measures of the neighbourhood food environment, and takeaway food purchasing practices and beliefs were associated with measures of public acceptability of takeaway management zones near schools. The strongest association was between reporting that the number of takeaways in the neighbourhood food environment was too high and supporting adoption. Less favourable beliefs about takeaways and takeaway food (such as their contribution to antisocial behaviour and litter, noise, and smells) were also consistently associated with greater support. Policy advocates could therefore emphasise the plausible societal benefits of these zones that extend beyond dietary health.

A sample of 16-17-year olds living in the UK agreed that takeaways have special offers aimed at them, are an important place for socialising, and sell low-cost food. These perspectives might be a reason why they also agreed that takeaway food consumption would continue even if there were fewer takeaways near schools. However, those in our study reported that if there were fewer takeaways near schools, then young people would not necessarily travel to takeaways further away to purchase food. This finding helps mitigate concerns about a possible displacement of the location of food purchasing practices following the adoption of these zones. The health of young people is often a core rationale for adoption of takeaway management zones around schools. How this group views these zones is not well understood. Investigating their perspectives about the scope and possible strengths and limitations of this intervention will be valuable for future development, monitoring, and evaluation.

## Supplementary Material

Supplemental Material

## Data Availability

Data from the International Food Policy Study cannot be made publicly available due to confidentiality considerations. Information about access to these data is available at https://foodpolicystudy.com/contact/. Data from the Ordnance Survey Points of Interest cannot be made publicly available due to licensing restrictions. Information about access to these data is available at https://digimap.edina.ac.uk/. The corresponding author can be contacted for assistance with processes to access these data.
