# Supplementary material for: Public acceptability of proposals to manage new takeaway food outlets near schools: cross-sectional analysis of the 2021 International Food Policy Study
Source: Cities Health. 2024 Apr 19;8(6):1094–107. doi: 10.1080/23748834.2024.2336311 (PMC11614041; doi:10.1080/23748834.2024.2336311)
Supplement: Supplemental Material [file RCAH_A_2336311_SM3494.pdf]

SUPPLEMENTARY MATERIAL

Table 1: Weighted summary statistics for adult respondents living in the UK (n=4196). Data were from Wave 5 of the International Food Policy Study, collected November-December 2021.

Measure	Category	N=4196[1]
Age (years - mean (SD))		48.5 (17.2)
Age group (years)	18-29	806 (19.2%)
	30-44	1026 (24.5%)
	45-59	1058 (25.2%)
	60 or over	1306 (31.1%)
Sex	Male	2052 (48.9%)
	Female	2144 (51.1%)
Ethnic group identified with	Majority	3702 (88.2%)
	Minority	470 (11.2%)
	Not stated	25 (0.6%)
Education level	Low	2089 (49.8%)
	Medium	888 (21.2%)
	High	1182 (28.2%)
	Not stated	37 (0.9%)
Ability to make ends meet	Very difficult	248 (5.9%)
	Difficult	650 (15.5%)
	Neither easy nor difficult	1494 (35.6%)
	Easy	1051 (25.0%)
	Very easy	699 (16.6%)
	Refuse to answer	27 (0.6%)
	Don't know	28 (0.7%)
Child under 18 years in household	Yes	1198 (28.6%)
	No	2990 (71.3%)
	Not stated	7 (0.2%)
Quartile (Q) of neighbourhood food outlet count	Q1 (0-9 outlets)	878 (24.2%)
	Q2 (20-25 outlets)	904 (24.9%)
	Q3 (26-55 outlets)	916 (25.2%)
	Q4 (56-582 outlets)	930 (25.6%)
	Unknown	568 (NA)
Number of meals purchased from takeaways in the past week	0	692 (16.5%)
	1	1136 (27.1%)
	2 or more	817 (19.4%)
	Question not asked [2]	1530 (36.5%)
	Don't know	10 (0.2%)
	Refuse to answer	11 (0.3%)
Perceived takeaway number appropriateness	Too many	1764 (42.0%)
	Too few	246 (5.9%)
	About the right number	1916 (45.7%)
	Refuse to answer	11 (0.3%)
	Don't know	259 (6.2%)
Takeaways:		
Usually sell food that is affordable	Agree	2036 (48.5%)
	Disagree	504 (12.0%)
	Neutral	1569 (37.4%)
	Refuse to answer	6 (0.2%)
	Don't know	80 (1.9%)
Usually sell food that is poor quality	Agree	1134 (27.0%)
	Disagree	948 (22.6%)
	Neutral	2015 (48.0%)
	Refuse to answer	4 (0.1%)
	Don't know	94 (2.2%)
Usually sell healthy food	Agree	559 (13.3%)
	Disagree	2244 (53.5%)
	Neutral	1285 (30.6%)
	Refuse to answer	5 (0.1%)
	Don't know	103 (2.5%)
Cause litter, noise and smells	Agree	2260 (53.9%)
	Disagree	486 (11.6%)
	Neutral	1349 (32.1%)
	Refuse to answer	7 (0.2%)

	Don't know	95 (2.3%)
Cause antisocial behaviour	Agree	1064 (25.3%)
	Disagree	1165 (27.8%)
	Neutral	1812 (43.2%)
	Refuse to answer	5 (0.1%)
	Don't know	150 (3.6%)
Contribute to the local economy	Agree	2412 (57.5%)
	Disagree	282 (6.7%)
	Neutral	1352 (32.2%)
	Refuse to answer	8 (0.2%)
	Don't know	142 (3.4%)
Support for management zone adoption [3]	Support	1944 (46.3%)
	Oppose	404 (9.6%)
	Neutral	1578 (37.6%)
	Refuse to answer	7 (0.2%)
	Don't know	263 (6.3%)
Perceived effectiveness of management zone adoption	Somewhat effective	1992 (47.5%)
	Mostly effective	564 (13.4%)
	Very effective	300 (7.1%)
	Not effective	992 (23.7%)
	Refuse to answer	9 (0.2%)
	Don't know	339 (8.1%)
Following management zone adoption:		
Takeaways would be replaced by healthier food outlets	Agree	1707 (40.7%)
	Disagree	611 (14.6%)
	Neutral	1641 (39.1%)
	Refuse to answer	4 (0.1%)
	Don't know	233 (5.5%)
It would be easier for schools to promote healthier food	Agree	1778 (42.4%)
	Disagree	736 (17.5%)
	Neutral	1469 (35.0%)
	Refuse to answer	4 (0.1%)
	Don't know	209 (5.0%)
Young people would eat takeaway food less often	Agree	1478 (35.2%)
	Disagree	999 (23.8%)
	Neutral	1472 (35.1%)
	Refuse to answer	7 (0.2%)
	Don't know	240 (5.7%)

[1] number (%) unless stated. May vary from total due to rounding after weighting. [2] Respondents who had not purchased at least one meal out-of-home in the week before survey completion were not asked questions about purchase location. [3] 'management zone' = takeaway management zones around schools, referred to as 'exclusion zones' elsewhere.

Table 2: Survey questions and response options in adult and youth surveys from Wave 5 of the International Food Policy Study, conducted November-December 2021.

Concept	Question wording [Adult]	Response options [Adult]	Question wording [Youth]	Response options [Youth]
Age	How old are you?	In years (continuous)	How old are you?	16 years<br>17 years
Sex	What sex were you assigned at birth, meaning on your original birth certificate?	Male<br>Female	What was your sex at birth?	Male<br>Female
Ethnicity	Which of the following best describes your ethnic or racial background?	White<br>Mixed / Multiple Ethnic Groups<br>Asian / Asian British<br>Black / African / Caribbean / Black British<br>Other Ethnic Group	Which of the following best describes your ethnic or racial background?	White<br>Mixed / Multiple Ethnic Groups<br>Asian / Asian British<br>Black / African / Caribbean / Black British<br>Other Ethnic Group
Level of education completed	What is the highest level of formal education that you have completed?	1 – 4 O levels/CSEs/GCSEs (any grades), Entry Level, Foundation Diploma, NVQ Level 1, Foundation GSVQ, Basic Skills<br><br>5+ O levels (passes)/CSEs (grade 1)/GCSEs (grades A*-C/9-4), School Certificate, 1 A level/2-3 AS levels/VCEs, Higher Diploma<br><br>No qualifications<br><br>NVQ Level 2, Intermediate GNVQ, City and Guilds Craft, BTEC First/General Diploma, RSA Diploma, Apprenticeship<br>2+ A levels/VCEs, 4+ AS levels, Higher School Certificate, Progression/Advanced Diploma, NVQ Level 3, Advanced GNVQ, City and Guilds Advanced Craft, ONC, OND, BTEC National, RSA Advanced Diploma<br><br>Foreign qualifications<br><br>Degree (for example BA, BSc), Higher Degree (for example MA, PhD, PGCE)<br><br>Professional qualifications (for example teaching, nursing, accountancy)<br><br>NVQ Level 4-5, HNC, HND, RSA Higher Diploma, BTEC Higher Level	-	-

Income adequacy	Thinking about your total monthly income, how difficult or easy is it for you to make ends meet?	Very difficult<br>Difficult<br>Neither easy nor difficult<br>Easy<br>Very easy<br>Don't know<br>Refuse to answer	Thinking about your total monthly income, how difficult or easy is it for you to make ends meet? [4]	Very difficult<br>Difficult<br>Neither easy nor difficult<br>Easy<br>Very easy<br>Don't know<br>Refuse to answer
Family income	-	-	Does your family have enough money to pay for things your family needs?	Not enough money<br>Barely enough money<br>Enough money<br>More than enough<br>Money<br>Don't know<br>Refuse to answer
Presence of child under 18 in household	How many of your children under the age of 18 live in your household (including stepchildren or adopted children)?	# of children [numeric: 0-10]<br>Don't know<br>Refuse to answer	-	-
Neighbourhood food outlet access	Please enter your postcode:<br><br>Postcodes help us to understand the food environment where you live. As a reminder, all information you provide will be kept strictly confidential and will never be shared	Postcode: [open text]<br>Don't know<br>Refuse to answer	-	-
Meals purchased from a takeaway in the past week	You said you had [#] meal(s) prepared outside the home in the past 7-days. How many of these meals did you get from each of the following locations?<br><br>Fast food / take-away / café (i.e., order from a counter, online, or by phone)	# of meals [numeric]<br>Don't know<br>Refuse to answer	Think about the last 7-days.<br><br>How many days did you have a meal (breakfast, lunch or evening meal) from restaurants, fast food or take-away places, food stands, or vending machines?	0 days (not at all)<br>1 day<br>2 days<br>3 days<br>4 days<br>5 days<br>6 days<br>7-days (every day)<br>Don't know<br>Refuse to answer
Consumption of takeaway food in past 24-hours	-	-	You just told us about the last 7-days. Now think about yesterday.<br><br>Did you have any of the following yesterday?<br><br>Fast food / take-away from a restaurant	Yes<br>No<br>Don't know<br>Refuse to answer

Perceived takeaway number appropriateness	Thinking about the number of takeaway shops you encounter on a day-to-day basis, do you think there are:	Too few<br>About the right number<br>Too many<br>Don't know<br>Refuse to answer	Thinking about the number of takeaways near your school, do you think there are:	Too few<br>About the right number<br>Too many<br>Don't know<br>Refuse to answer
Belief about takeaway food healthiness	Please tell us whether you agree or disagree with the following:<br><br>Takeaways usually sell healthy food.	Agree<br>Neutral<br>Disagree<br>Don't know<br>Refuse to answer	We'd like to know what you think about takeaways (answer true or false to the following statements):<br><br>Takeaways usually sell healthy food	True<br>False<br>Don't know<br>Refuse to answer
Belief about takeaway food quality	*Preamble as above*<br><br>Takeaways usually sell food that is poor quality.	Agree<br>Neutral<br>Disagree<br>Don't know<br>Refuse to answer	-	-
Belief about the monetary cost of takeaway food	*Preamble as above*<br><br>Takeaways usually sell food that is affordable.	Agree<br>Neutral<br>Disagree<br>Don't know<br>Refuse to answer	*Preamble as above*<br><br>Takeaways usually sell food that is low-cost	True<br>False<br>Don't know<br>Refuse to answer
Belief about litter, noise and smells from takeaways	*Preamble as above*<br><br>Takeaways cause litter, noise and smells	Agree<br>Neutral<br>Disagree<br>Don't know<br>Refuse to answer	-	-
Belief about takeaways and antisocial behaviour	*Preamble as above*<br><br>Takeaways cause antisocial behaviour	Agree<br>Neutral<br>Disagree<br>Don't know<br>Refuse to answer	-	-
Belief about the contribution of takeaways to the local economy	*Preamble as above*<br><br>Takeaways contribute to the local economy	Agree<br>Neutral<br>Disagree<br>Don't know<br>Refuse to answer	-	-
Belief about takeaway special offers	-	-	*Preamble as above*<br><br>Takeaways have special offers for young people	True<br>False<br>Don't know<br>Refuse to answer
Belief about takeaways as a social venue	-	-	*Preamble as above*<br><br>Takeaways are an important place for young people to hang out	True<br>False<br>Don't know<br>Refuse to answer

Impact of adoption of a takeaway management zone around a school	-	If there were fewer takeaways near schools (select all that apply):<br><br>Young people would be more likely to eat lunch in the school canteen<br><br>Young people would buy unhealthy food from other types of food outlets<br><br>Young people would eat takeaway food less often<br><br>Young people would travel to takeaways further away from school<br><br>Young people would have takeaway food delivered to school<br><br>Don’t know<br>Refuse to answer	Boxes checked or unchecked (i.e. agree or disagree)

Table 3: Model One; Adjusted Odds Ratios (OR) and 95% Confidence Intervals (CI) of sociodemographic characteristics, measures of the neighbourhood food environment, and takeaway food purchasing practices associated with public acceptability of takeaway management zones among adults living in Great Britain (n=3323)[1]. Data were from Wave 5 of the International Food Policy Study, collected November-December 2021, analysed using logistic regression.

	Support for adoption		Perceived effectiveness		Healthier outlets able to open		Healthier school food promotion		Less takeaway food consumption	
Measure [2]	OR	95% CI	OR	95% CI	OR	95% CI	OR	95% CI	OR	95% CI
Age group (years); 18-29 = ref										
30-44	**1.45**	**1.10, 1.90**	**0.51**	**0.37, 0.71**	**1.39**	**1.07, 1.81**	1.05	0.80, 1.36	**1.39**	**1.06, 1.82**
45-59	**2.37**	**1.79, 3.15**	**0.42**	**0.30, 0.58**	**1.35**	**1.02, 1.79**	**1.34**	**1.02, 1.77**	**1.59**	**1.20, 2.13**
60 or over	**3.53**	**2.65, 4.70**	**0.47**	**0.34, 0.65**	**1.50**	**1.14, 1.99**	**1.88**	**1.43, 2.48**	**1.91**	**1.43, 2.55**
Sex; Female (vs Male)	1.14	0.96, 1.34	1.08	0.91, 1.29	**1.19**	**1.01, 1.40**	**1.19**	**1.01, 1.39**	0.96	0.81, 1.12
Ethnic group identified with; Minority (vs Majority)	1.27	0.96, 1.68	**1.57**	**1.11, 2.24**	1.30	0.98, 1.72	**1.44**	**1.09, 1.91**	**1.45**	**1.09, 1.92**
Education level; Low = ref										
Medium	**1.32**	**1.08, 1.62**	1.02	0.82, 1.26	1.00	0.82, 1.21	1.05	0.86, 1.28	1.11	0.90, 1.35
High	**1.63**	**1.35, 1.96**	1.15	0.94, 1.40	0.98	0.82, 1.17	1.17	0.98, 1.40	1.16	0.97, 1.40
Ability to make ends meet; Easy (vs Not easy)	**1.46**	**1.24, 1.73**	1.16	0.97, 1.39	1.15	0.98, 1.36	1.17	0.99, 1.38	**1.19**	**1.01, 1.41**
Child under 18 years in household; Yes (vs No)	0.91	0.73, 1.13	**1.51**	**1.18, 1.93**	0.94	0.77, 1.16	1.17	0.95, 1.44	1.04	0.84, 1.29
Quartile (Q) of neighbourhood food outlet access (count); Q1 (0-9 outlets) = ref										
Q2 (10-25 outlets)	0.89	0.71, 1.12	1.23	0.97, 1.57	1.03	0.83, 1.29	1.01	0.81, 1.26	0.98	0.78, 1.23
Q3 (26-55 outlets)	1.00	0.79, 1.25	1.08	0.85, 1.38	1.06	0.85, 1.33	1.06	0.85, 1.33	1.17	0.93, 1.47
Q4 (56-581 outlets)	0.91	0.71, 1.15	1.11	0.86, 1.43	1.05	0.83, 1.32	1.15	0.91, 1.45	1.19	0.94, 1.51
Meals purchased from takeaways in the past week (count); 0 = ref										
1	0.86	0.70, 1.05	1.01	0.81, 1.27	1.11	0.91, 1.36	1.12	0.92, 1.37	1.17	0.96, 1.44
2 or more	0.80	0.62, 1.01	1.09	0.83, 1.43	1.24	0.98, 1.57	1.06	0.83, 1.34	1.14	0.89, 1.45

[1] All results are adjusted for all other measures listed. Bold = significant at the P<0.05 level. [2] 'ref' = reference category.
